# Large Responsivity of Graphene Radiation Detectors with Thermoelectric Readout: Results of Simulations

**DOI:** 10.3390/s20071930

**Published:** 2020-03-30

**Authors:** August Yurgens

**Affiliations:** Department of Microtechnology and Nanoscience (MC2), Chalmers University of Technology, SE-412 96 Gothenburg, Sweden; yurgens@chalmers.se

**Keywords:** graphene, thermoelectric effects, solid-state detectors

## Abstract

Simple estimations show that the thermoelectric readout in graphene radiation detectors can be extremely effective even for graphene with modest charge-carrier mobility ∼1000 cm${}^{2}$/(Vs). The detector responsivity depends mostly on the residual charge-carrier density and split-gate spacing and can reach competitive values of ∼$10^{3}$–$10^{4}$ V/W at room temperature. The optimum characteristics depend on a trade-off between the responsivity and the total device resistance. Finding out the key parameters and their roles allows for simple detectors and their arrays, with high responsivity and sufficiently low resistance matching that of the radiation-receiving antenna structures.

## 1. Introduction

The graphene radiation detectors promise to be fast and sensitive devices in a broad frequency band from sub-THz- to infrared spectrum of electromagnetic radiation, operational from ambient [1] to cryo temperatures [2]. A negligibly small thermal mass of a typical graphene radiation absorber guarantees a very short response time of the detector [3,4,5,6,7,8]. Several readout mechanisms in graphene detectors have been identified—bolometric [9], thermoelectric (TEP) [4], ballistic [10], based on noise thermometry [11], and electron-plasma waves [12], commonly called the Dyakonov-Shur (D-S) mechanism [13,14]. However, resistivity of graphene changes significantly with temperature only in graphene samples with induced bandgap and only at low temperature. In the noise thermometry, the electronic temperature is obtained from first principles but the measurement setups are complex and therefore impractical. Both ballistic and D-S mechanisms require very high mobility samples, in most cases obtained by laborious encapsulation of graphene in between hexagonal boron nitride (hBN) flakes. hBN of sufficiently high quality is unique and apparently available from only one laboratory in the World [15].

The TEP readout favorably stands out from the rest because of its simplicity, room-temperature operation, no electrical bias and therefore no 1/*f* noise, scalable fabrication using CVD graphene, and undemanding electrical contacts. This combination of detector properties is particularly important for the fabrication of large detector arrays. The effectiveness of this readout stems from a high value of the Seebeck coefficient (S∼100μV/K) [16,17] and easy control over the charge-carrier density and sign in graphene. An electrostatically induced *p-n* junction gives an all-set access to the electronic temperature *T* in graphene, thereby meeting the main requirement for radiation detectors. The temperature increase caused by incoming radiation is high because of a weak electron-phonon (e-ph) coupling in graphene [18,19]. Combination of the weak e-ph coupling with large *S* gives a strong foundation for building radiation-sensitive devices.

Among practical devices reported in the literature, graphene detectors with TEP readout experimentally demonstrated quite high responsivity 100–1000 V/W and low noise-equivalent power $NEP∼20–200\;\mathrm{pW}/\sqrt{\mathrm{Hz}}$ [4,7,20,21,22,23]. The responsivity ℜ of detectors with TEP readout is usually 10–100 times higher than in those based on graphene field-effect transistors (GFETs), unless GFETs have very high mobility [12]. The spread of device characteristics in the literature requires some qualitative understanding of key parameters that have the major effect on the detector performance. Here, the limiting values of ℜ have been estimated by using earlier experimental data on electron cooling efficiency in graphene [18,24]. These estimations give basic guidelines on optimizing detectors with simple geometry and graphene of undemanding quality.

## 2. Model

**Figure 1:**
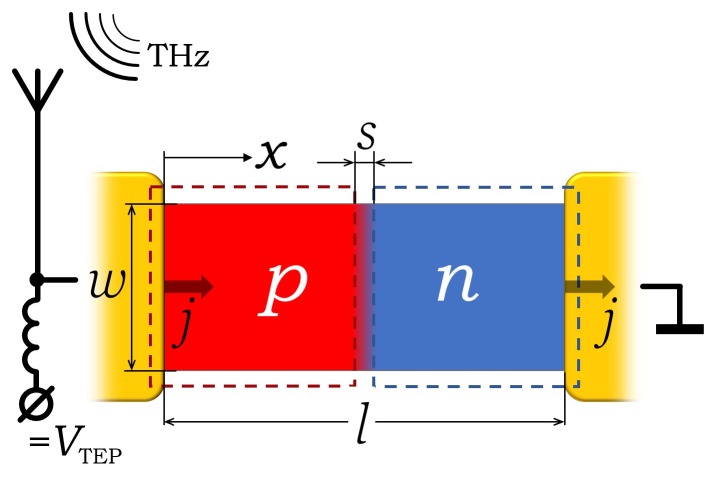
The model geometry of graphene detector with a *p-n*-junction in the center. The *p*- and *n* regions are induced electrostatically by a split gate with two parts (dashed rectangles) separated by the distance *s*. A current with linear density *j* flows along the strip.

The model geometry is shown in Figure 1. A graphene strip of length *l* and width *w* is subdivided into *p*- and *n* regions. The strip rests on a substrate with an infinitely high thermal conductivity. The electrical current with the linear density *j* flows in *x* direction from the source- to drain electrodes made of thick metal films. The temperature $T_{0}$ of the substrate, electrodes, and phonons in graphene is assumed to be constant. The electrons in graphene are heated by the current and cooled by phonons through the electron-phonon interaction. The heating- and resulting temperature distribution T(x) are highly non-uniform because of the spatially varying doping profile.

The charge-density (doping) profile $n_{d}\left(x\right)$ is approximated by:

(1) $$n_{d}\left(x\right)=n_{\max}\left[F\left(\frac{x-l/2+s}{\delta }\right)-F\left(\frac{-x+l/2+s}{\delta }\right)\right],$$

where F(u)=1/[1+exp(u)] is the Fermi function, $n_{\max}$ is the maximum induced doping, *s* is the separation between the gates, and δ determines smearing of the profile due to fringing of the electric field at the gates edges; δ∼ the gate-dielectric thickness. In the conceivably possible case of chemical doping, δ would correspond to the lateral gradient of dopant concentration. For some brevity in equations, $n_{d}$ has sign, reflecting the sign of charge carriers in the *p*- and *n* regions.

The region with the smallest $n_{d}$, that is, the *p-n* junction, has the lowest electrical conductance σ, which changes very little with temperature and therefore is taken to depend on *x* only (Equation (1)):

(2) $$\sigma \left(n_{d}\right)=\mu \left|e\right|\sqrt{n_{d}^{2}+n_{0}^{2}},$$

where μ is the charge-carrier mobility, *e* is the elementary charge, and $n_{0}$ is the residual charge density,

(3) $$n_{0}\left(T\right)=n_{00}+\beta T^{2},$$

where $n_{00}$ is the part resulting from the charge puddles [25] and the second term is due to smearing of the Fermi energy by temperature puddles [25] and temperature with $\beta =\left(\pi /6\right)k_{B}^{2}/{\left(\hbar v_{F}\right)}^{2}$ [26]; $k_{B}$, ℏ, and $v_{F}$ are the Boltzmann- and Planck constants and the Fermi velocity, respectively. This model is described by the one-dimensional heat equation:

(4) $$-\frac{\partial }{\partial x}\left(\kappa_{e}\frac{\partial T}{\partial x}\right)=\frac{j^{2}}{\sigma }-jT\frac{\partial S}{\partial x}-\alpha_{i}\left(T^{i}-T_{0}^{i}\right),$$

where $\kappa_{e}=L_{0}\sigma T$ is the electronic sheet thermal conductivity and $L_{0}$ is the Wiedemann–Franz constant. The three terms on the right-hand side of the equation correspond to the Joule heating, Peltier effect and electron-phonon cooling, respectively. The Seebeck coefficient *S* in graphene is assumed to obey Mott’s equation in the whole temperature range.

(5) $$S=\frac{\pi^{2}}{3}\frac{k_{B}}{\left|e\right|}k_{B}T\frac{\partial \ln\sigma }{\partial n_{d}}\frac{\partial n_{d}}{\partial E_{F}}.$$

where $E_{F}$ is the Fermi energy. The heat transfer to the phonon system is described by the last term in Equation (4). The exponent i=3 or i=4 at temperatures above or below the Bloch-Gruneisen temperature $T_{\mathrm{BG}}$, respectively, $\alpha_{3}\approx \left(0.1–0.12\right)n_{d}\;[\times 10^{-15}\;W/K^{3}]$ [18] and $\alpha_{4}\approx 0.5\;\mathrm{mW}/(m^{2}K^{4})$ [24]. The second term in Equation (4) is much larger than the third one at low temperature; to know the exact value of $\alpha_{4}$ is therefore not important.

Numerically solving Equation (4) gives T(x), the TEP voltage, and the total Joule dissipation for any bias current *j*. The current in real detectors is induced by the incoming radiation and is periodically varying with time *t*: $j\left(t\right)=j_{0}\sin\left(\omega t\right)$. For ω≪2π/τ, where τ<50 fs is the electron-heating time [8], the responsivity ℜ can be found by averaging the voltage and Joule power over one period of the ac bias:

(6) $$\begin{array}{ccc} V_{\mathrm{TEP}} & = & \langle \int_{0}^{l}S\left(x\right)\nabla T\left(x\right)dx\rangle \end{array}$$

(7) $$\begin{array}{ccc} P_{\mathrm{tot}} & = & \langle j^{2}\int_{0}^{l}\frac{wdx}{\sigma (x)}\rangle \end{array}$$

(8) $$\begin{array}{ccc} ℜ & = & V_{\mathrm{TEP}}/P_{\mathrm{tot}}. \end{array}$$

## 3. Results

**Figure 2:**
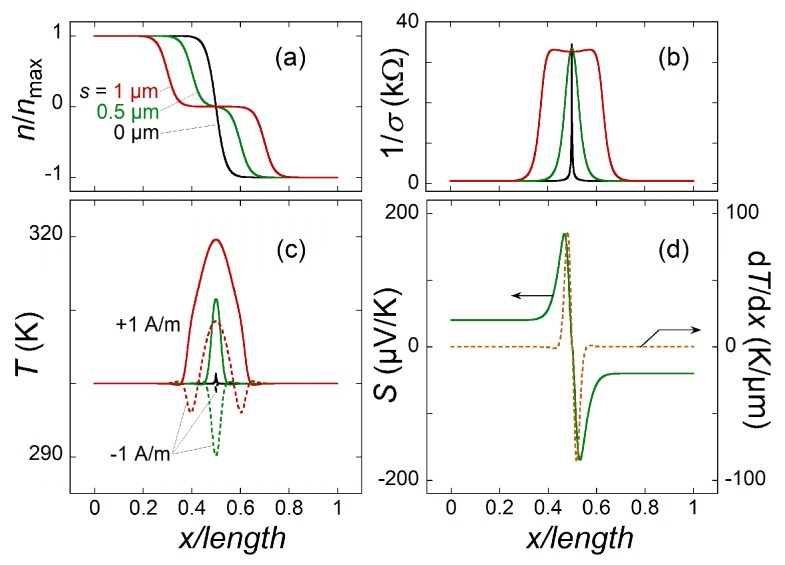
(**a**) The doping profile. (**b**) The local sheet resistance. (**c**) The temperature distributions for j=+1 and −1 A/m (solid and dashed curves, respectively). The Peltier effect is a significant source of the temperature variations. (**d**) The temperature gradient (orange) and Seebeck coefficient calculated from (**a**,**c**). Only narrow region around x=l/2 contributes to the output signal $V_{\mathrm{TEP}}$. The curves in (**a**–**c**) correspond to s=0, 0.5, and 1 μm, (**d**) to only s=0.5μm; $n_{00}=10^{11}\;1/{\mathrm{cm}}^{2}$ for all panels; j=+1 A/m in (**c**,**d**). All panels correspond to $T_{0}=300$ K.

The following parameters were used in the calculations: $l\times w=5\times 5\;\mu m^{2}$, δ = 0.1 μm, s=0…1μm (see Figure 1), $n_{\max}=1\times 10^{13}\;1/{\mathrm{cm}}^{2}$, $n_{00}=\left(1\ldots 100\right)\times 10^{10}\;1/{\mathrm{cm}}^{2}$, $\mu =10^{3}$ or $10^{4}\;{\mathrm{cm}}^{2}/\left(\mathrm{Vs}\right)$, $T_{0}=$ (4, 100, 200, 300 K), and $j_{0}=\left(0.01\ldots 1\right)$ A/m. The total power (Equation (7)) corresponding to this range of $j_{0}$ is from 2 pW to 240 nW for s=0 and 1 μm, respectively, assuming $\mu =10^{3}\;{\mathrm{cm}}^{2}/\left(\mathrm{Vs}\right)$ and $n_{00}=10^{11}\;1/{\mathrm{cm}}^{2}$ (see Figure 2). $P_{\mathrm{tot}}$ is ten times smaller for $\mu =10^{4}\;{\mathrm{cm}}^{2}/\left(\mathrm{Vs}\right)$. For each $n_{00}$ and $T_{0}$, $j_{0}$ is chosen to be sufficiently small, to restrict the maximum temperature rise at the *p-n* junction to less than 10% of $T_{0}$. Values of $T_{0}$ are picked in correspondence with the limiting cases of $T_{0}<T_{\mathrm{BG}}$ and $T_{0}>T_{\mathrm{BG}}$. The results of calculations for some combination of these parameters are shown in Figure 2.

Because of a low σ at zero doping, the Joule heating is maximal in the center of the graphene strip. It is seen that T(x) (Figure 2c) changes in agreement with the $n_{d}\left(x,s\right)$ curves (Figure 2a). The wider the region of zero doping the wider the T(x). The change from heating ($T\left(x\right)>T_{0}$) to cooling ($T\left(x\right)<T_{0}$) occurs because of the Peltier effect, which is a substantial source of temperature variation. The temperature gradient and Seebeck coefficient are shown in (Figure 2d). The integral of their product gives the overall TEP signal. Clearly, only the parts of the strip where dT/dx≠0 contribute to the signal and it is favorable to have a smeared doping profile, that is, larger δ and/or *s* (see below).

**Figure 3:**
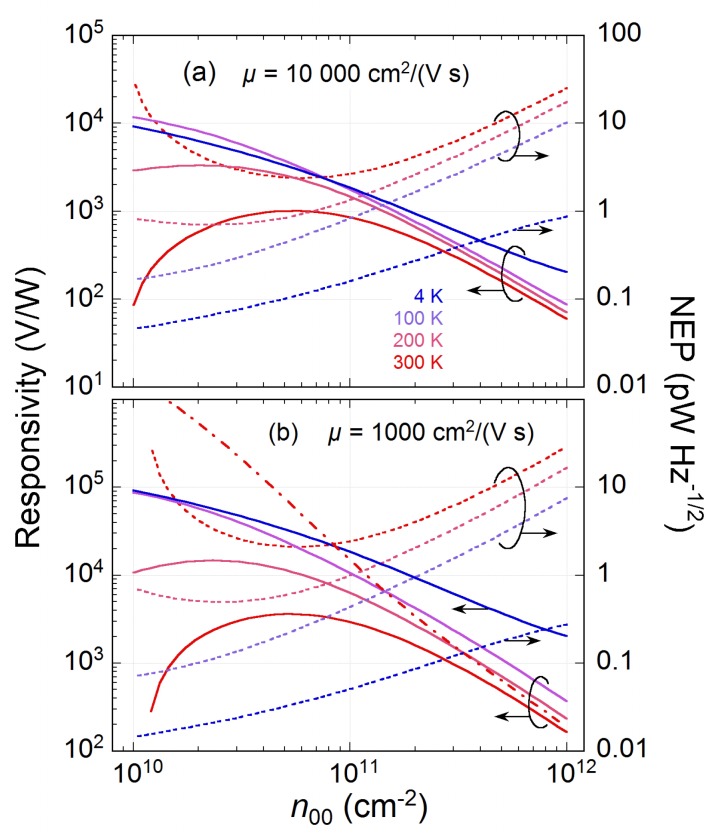
The responsivity ℜ (left ordinate, solid lines) and noise equivalent power NEP (right, dotted lines) versus residual charge density due to charge puddles ($n_{00}$) for different ambient temperatures. δ=0.1, s=0.5μm, $\mu =10^{4}$ (**a**) and $10^{3}\;{\mathrm{cm}}^{2}/\left(\mathrm{Vs}\right)$ (**b**). Ignoring the temperature dependence of $n_{0}$ (Equation (3)) gives too high $ℜ>10^{6}$ V/W (dash-dotted line in (**b**)).

Figure 3 shows the effect of probably the most important parameter, $n_{00}$, on ℜ at different $T_{0}$ and for $\mu =10^{3}$ and $10^{4}\;{\mathrm{cm}}^{2}/\left(\mathrm{Vs}\right)$. The responsivity significantly increases upon lowering $n_{00}$, reaching a competitive value of $\geq 10^{4}\;V/W$ for graphene with $\mu =10^{3}\;{\mathrm{cm}}^{2}/\left(\mathrm{Vs}\right)$. For a ten times higher μ, ℜ decreases roughly ten times. However, the advantage of having graphene with high mobility is ten times smaller overall resistance, $R_{\mathrm{tot}}∼1$ and 10 kΩ for $\mu =10^{4}$ and $10^{3}\;{\mathrm{cm}}^{2}/\left(\mathrm{Vs}\right)$, respectively. This is important for impedance matching between graphene and a radiation-collecting antenna. Also, the temperature dependence of the residual charge density $n_{0}$ (Equation (3)) is significant. Without it, the responsivity gets unrealistically high $ℜ>10^{6}$ V/W (see the dash-dotted line in Figure 3b). By dividing the thermal noise voltage variance per 1 Hz of bandwidth ($\sqrt{4k_{B}TR_{\mathrm{tot}}}$) by the responsivity, the noise equivalent power (NEP) can be calculated.

**Figure 4:**
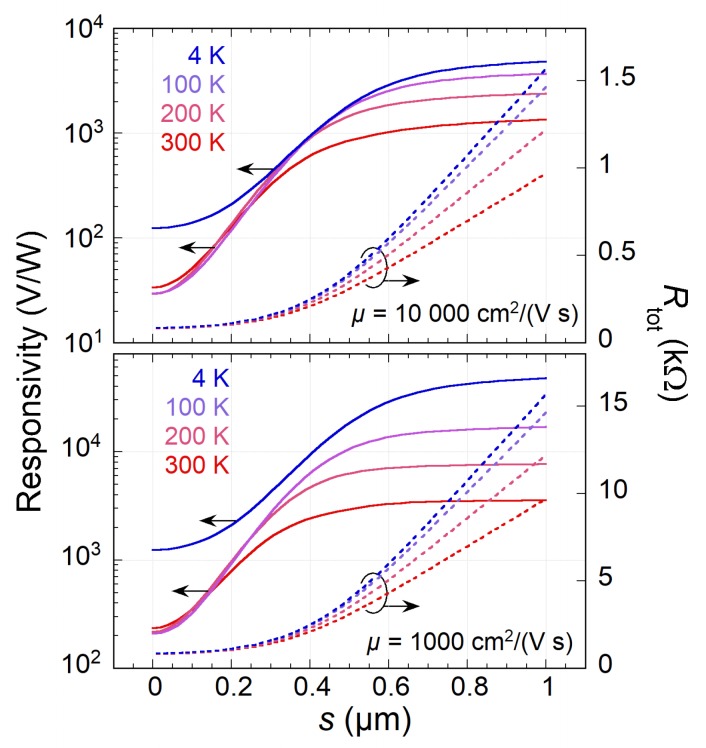
The responsivity ℜ (left ordinate) and overall resistance $R_{tot}$ (right) versus the split-gate separation *s* for δ=0.1μm, $\mu =10^{4}$ (**Top**) and $10^{3}\;{\mathrm{cm}}^{2}/\left(\mathrm{Vs}\right)$ (**Bottom**), and different $T_{0}=4$, 100, 200, and 300 K.

Next, the effect of split-gate separation *s* is shown in Figure 4. The smearing of T(x) increases with *s* and is followed by a dramatic increase of ℜ at the expense of high $R_{\mathrm{tot}}$. At a relatively large *s*, the graphene channel is distinctly divided into three parts, *p*, neutral, and *n* (see Figure 2a), which is effectively equivalent to the *p-n* junction extending in space. The increase of responsivity is then largely due to the increased Joule dissipation in the neutral region of graphene.

## 4. Discussion

As has been demonstrated above, $n_{00}$ is the main parameter governing ℜ, which can be as high as $3\times 10^{3}$ V/W at T=300 K for $n_{00}\approx 5\times 10^{10}\;1/{\mathrm{cm}}^{2}$ and $\mu =10^{3}\;{\mathrm{cm}}^{2}/\left(\mathrm{Vs}\right)$ (see Figure 3). Of course, the majority of practical devices have larger $n_{00}>10^{11}\;1/{\mathrm{cm}}^{2}$. Even then, $ℜ∼10^{3}$, which is in agreement with experiments [21]. However, it has recently been found that in graphene grown on SiC, the residual doping can be very small, close to $n_{0}∼10^{10}\;1/{\mathrm{cm}}^{2}$ [27]. The temperature is then the main factor affecting $n_{0}$, resulting in a strong temperature dependence of graphene resistance R(T), which, in turn, allows for the development of a low-temperature bolometer mixer [6]. Note, that if the TEP readout were used instead of the bolometric one, a high $ℜ∼10^{5}$ V/W could possibly be reached at much higher $T_{0}∼100$ K (see Figure 3).

**Figure 5:**
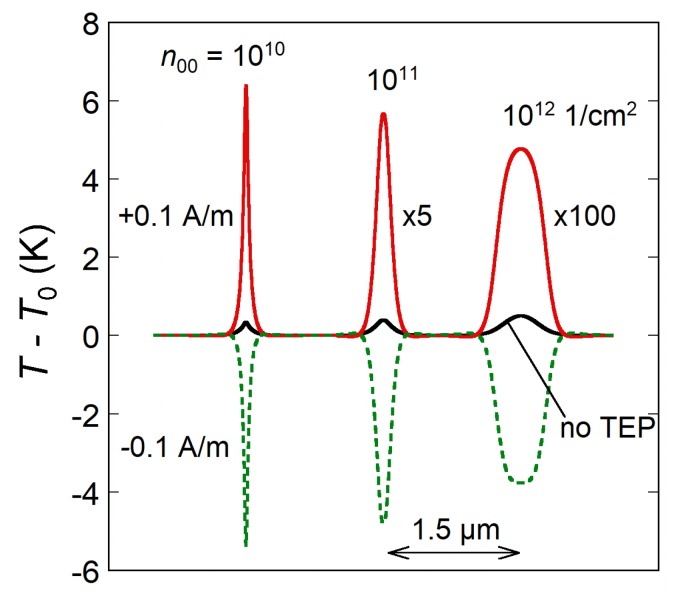
The temperature change $T\left(x\right)-T_{0}$ corresponding to heating (**Red**) and cooling (**Green**) of the *p-n* junction by ±0.1–A/m current for $n_{00}=10^{10},10^{11},\;\mathrm{and}\;10^{12}\;1/{\mathrm{cm}}^{2}$ (left to right). The curves are shifted horizontally for clarity. $\mu =1000\;{\mathrm{cm}}^{2}/\left(\mathrm{Vs}\right)$, δ=0.1μm, s=0.5μm, and $T_{0}=100$ K. The black curves correspond to the Joule heating only, without the Peltier effect.

The Peltier effect is obviously dominant in the heating of graphene *p-n* junctions, especially for sufficiently uniform graphene with $n_{00}\leq 10^{11}\;1/{\mathrm{cm}}^{2}$. Figure 5 shows the temperature distribution for different $n_{00}$. For comparison, there are curves corresponding to the thermoelectric effects switched off. It is noteworthy that the temperature in a *p-n* junction would change much more dramatically than if only Joule heating were considered. This is also clear from the comparison between the first (Joule) and the second (Peltier) terms in Equation (4). The latter is typically 10–100 times larger than the former at high $T_{0}$. This prompts for using *p-n* junctions in graphene as thermal sources of infrared light [28].

The TEP readout, because of its open-circuit condition, is expected to be limited by the thermal Johnson–Nyquist noise only, contrary to other types of readout using some bias current. In the presence of bias current, the 1/*f* noise starts to dominate. The 1/*f* noise in graphene is rather high and extends to frequencies $∼10^{5}$ Hz [29], which can be a problem for detector systems with mechanical beam choppers. Figure 3 shows that the NEP does not change much with increasing μ - the reduced responsivity is compensated by a lower thermal noise because of a smaller $R_{\mathrm{tot}}$ at high μ. For a typical $n_{00}∼2–4\times 10^{11}$ 1/cm${}^{2}$, NEP lies between 1 and 10 pW/Hz${}^{1/2}$ at high temperature. This is at least ten times better than the NEPs of other types of uncooled direct detectors [30]. These estimations are also in agreement with the recent experimental works on TEP readout, for example, Reference [4,20,21].

Even in GFET-based detectors, the TEP readout mechanism can contribute with a substantial, if not dominating, signal [2,31]. The detection signal in GFETs is proportional to the transfer characteristic of GFET, that is, to $d\ln\left(\sigma \right)/dV_{g}$, with $V_{g}$ being the gate voltage. The same combination of values is involved in Mott’s equation (Equation (5)), given that $n_{d}\propto V_{g}$. This makes these detection mechanisms difficult to tell apart and/or totally exclude the TEP contribution to the signal (see e.g., References [12,32] for recent experimental efforts). Indeed, the top gate subdivides the graphene channel into three regions with conceivably different doping: *p-p’-p* or *n-n’-n*. If graphene globally is close to the charge neutrality point, the doping of different regions can have opposite signs, *p-n-p* or *n-p-n*, thereby creating two *p-n* junctions in series. When the AC current, which is injected via the gate, flows predominantly towards either the source or drain, only one of the *p-n* junctions will be heated by the current. This breaks the symmetry and gives rise to an uncompensated TEP signal. The farther apart the *p-n* and *n-p* junctions (i.e., the wider the gate) the more asymmetric is the current through the junctions and the higher the responsivity can be expected [33]. For graphene with very high mobility [12], it is, however, tempting to speculate that the TEP- and D-S detection mechanisms might become mixed together, possibly resulting in responsivity amplification. This however requires a thorough theoretical analysis.

The present work assumed that the current was applied through the metal electrodes while ignoring their contact resistances to graphene. The contact resistance can be sufficiently low for the edge contacts, which are possible for graphene encapsulated in both hBN [34] and Parylene [20,35], the latter being important for scaling up the device fabrication. Also, the contact resistance can be effectively reduced by increasing the perimeter of contact even for a non-encapsulated graphene [36]. Finally, the capacitive coupling of antennas to graphene, where the contact resistance is not so important for the open-circuit TEP readout, has recently been realized [21,22].

## 5. Conclusions

The effectiveness of the thermoelectric readout mechanism in graphene radiation detectors has been estimated for a few key parameters, assuming a simple device geometry. The residual charge density and sharpness of the *p-n* junction are the main parameters that affect the detector performance most. In all cases, there is a trade-off between the responsivity and the total device resistance. Concluding, the thermoelectric readout in graphene radiation detectors represents a very competitive platform for building simple and sensitive direct detectors of radiation and arrays of them.
